# Passive electroreception in bottlenose dolphins (*Tursiops truncatus*): implication for micro- and large-scale orientation

**DOI:** 10.1242/jeb.245845

**Published:** 2023-11-30

**Authors:** Tim Hüttner, Lorenzo von Fersen, Lars Miersch, Guido Dehnhardt

**Affiliations:** ^1^ Institute for Biosciences, University of Rostock, Albert-Einstein-Strasse 3, 18059 Rostock, Germany; ^2^ Behavioral Ecology and Conservation Lab, Nuremberg Zoo, Am Tiergarten 30, 90480 Nuremberg, Germany

**Keywords:** Sensory ecology, Toothed whales, Sensory systems, Vibrissal crypts, Benthic feeding, Geomagnetic orientation

## Abstract

For the two dolphin species *Sotalia guianensis* (Guiana dolphin) and *Tursiops truncatus* (bottlenose dolphin), previous research has shown that the vibrissal crypts located on the rostrum represent highly innervated, ampullary electroreceptors and that both species are correspondingly sensitive to weak electric fields. In the present study, for a comparative assessment of the sensitivity of the bottlenose dolphin's electroreceptive system, we determined detection thresholds for DC and AC electric fields with two bottlenose dolphins. In a psychophysical experiment, the animals were trained to respond to electric field stimuli using the go/no-go paradigm. We show that the two bottlenose dolphins are able to detect DC electric fields as low as 2.4 and 5.5 µV cm^−1^, respectively, a detection threshold in the same order of magnitude as those in the platypus and the Guiana dolphin. Detection thresholds for AC fields (1, 5 and 25 Hz) were generally higher than those for DC fields, and the sensitivity for AC fields decreased with increasing frequency. Although the electroreceptive sensitivity of dolphins is lower than that of elasmobranchs, it is suggested that it allows for both micro- and macro-scale orientation. In dolphins pursuing benthic foraging strategies, electroreception may facilitate short-range prey detection and target-oriented snapping of their prey. Furthermore, the ability to detect weak electric fields may enable dolphins to perceive the Earth's magnetic field through induction-based magnetoreception, thus allowing large-scale orientation.

## Teaser

**Highlighted Article:** Bottlenose dolphins can detect electric fields in water and could be able to use this information for short-range prey detection as well as large-scale orientation.

## INTRODUCTION

Electroreception ­– the ability to perceive weak electric fields – is found almost exclusively in aquatic or semi-aquatic species and can be either active or passive. While weakly electric fish (Gymnotiformes and Mormyriformes) generate electric discharges (electric organ discharges, EODs) with specialized electric organs for active electrolocation or electrocommunication (von der Emde, 1999; Kramer, 1996; Lissmann, 1951; Lissmann and Machin, 1958), passive electroreceptive species can only detect electric signals from their environment. Weak bioelectric fields are a reliable short-range source of information for passive electroreceptive animals as all organisms produce electric direct current (DC) fields in the water owing to ion flow during osmoregulation and general cell activity, for example (Bedore and Kajiura, 2013; Kalmijn, 1972). The standing DC field can be modulated by a low-frequency alternating current (AC) potential, which is caused by respiratory muscle activity (Wilkens and Hofmann, 2008). Bioelectric fields facilitate essential activities such as finding prey (Kalmijn, 1966), locating a mating partner (Tricas et al., 1995; Tricas and Sisneros, 2004) and avoiding predators (Kempster et al., 2013; Sisneros et al., 1998). In addition, passive electroreception generally has the potential for large-scale orientation based on the perception of geomagnetic fields through electromagnetic induction in marine habitats (Courtney et al., 2015; Jungerman and Rosenblum, 1980; Kalmijn, 1981).

Passive electroreception has evolved independently across different taxa. It is found in almost all non-teleost fish, four groups of teleost fish, and some amphibians (Newton et al., 2019). Among mammals, passive electroreception has experimentally been demonstrated in the monotreme species *Tachyglossus aculeatus* (Gregory et al., 1989b; Proske et al., 1998) and *Ornithorhynchus anatinus* (Fjällbrant et al., 1998; Gregory et al., 1989a; Iggo et al., 1992; Manger and Pettigrew, 1995; Manger et al., 1996; Scheich et al., 1986), as well as in the Guiana dolphin (*Sotalia guianensis*; Czech-Damal et al., 2012) and the bottlenose dolphin (*Tursiops truncatus*; Hüttner et al., 2022). For the platypus (*Ornithorhynchus anatinus*), behavioral studies revealed detection thresholds of 25–50 µV cm^−1^ (Fjällbrant et al., 1998; Manger and Pettigrew, 1995; Scheich et al., 1986), whereas the Guiana dolphin still detected weak electric fields of 4.6 µV cm^−1^ (Czech-Damal et al., 2012). In the Guiana dolphin, exclusion tests identified modified vibrissal follicles, located in two rows on both sides of the upper rostrum, as electroreceptors. The dolphins lose the vibrissal hairs shortly after birth and only the hairless vibrissal crypts remain. Contrary to earlier thermographic investigations (Mauck et al., 2000), which suggested, except for the missing hair, intact vibrissal follicle–sinus complexes (Rice et al., 1986) in the Guiana dolphin, the vibrissal crypts lack most of the classic follicle structures. Instead, each crypt consists of an ampulla-shaped invagination of the epidermal integument, densely innervated by 300 axons derived from infraorbital branches of the trigeminal nerve (Czech-Damal et al., 2012). This overall structure closely resembles the structure of other ampullary electroreceptors such as the mucous gland electroreceptors of the platypus or the ampullae of Lorenzini of sharks and rays (Czech, 2007; Czech-Damal et al., 2012; Dehnhardt et al., 2020; Manger and Pettigrew, 1996; Metcalf, 1915; Murray, 1974).

The electroreceptive capabilities of the Guiana dolphin support the benthic foraging strategy described for this species. Furthermore, analyses of the stomach contents of this Brazilian dolphin species suggest that demersal fish represent their main prey (Di Beneditto and Siciliano, 2007; Gurjão et al., 2003). Field studies on foraging Guiana dolphins found that after diving, dolphins often breach amidst clouds of mud with their bodies covered by benthic sediment. This indicates that they search for bottom-dwelling fish, which they dig for in the sediment (Rossi-Santos and Wedekin, 2006). To do so, a dolphin might use its passive electrosensory capability at short-range when vision and/or echolocation is restricted (Czech-Damal et al., 2012).

Using the benthos as a foraging niche is suggested for several odontocete species (Dehnhardt et al., 2020), including the bottlenose dolphin, for which a strategy known as ‘crater feeding’ has been described (Rossbach and Herzing, 1997). Recent studies revealed that the vibrissal crypts on the rostrum of neonate bottlenose dolphins still show many of the structural characteristics of vibrissal follicle–sinus complexes in terrestrial mammals (Gerussi et al., 2020; Hüttner et al., 2022). However, those in adult bottlenose dolphins show profound transformations and ultimately resemble the ampullary vibrissal crypts described for the electroreceptive Guiana dolphin (Hüttner et al., 2022). The structural transformation of vibrissal crypts in adult bottlenose dolphins to an ampullary system is accompanied by an innervation of each crypt by 245 myelinated axons, a value that comes close to that determined for vibrissal crypts in the Guiana dolphin (Czech-Damal et al., 2012). Compared with terrestrial mammals, the innervation of the vibrissal crypts of dolphins is similar to or even surpasses that of the follicle–sinus complexes of species considered to be richly innervated (Marotte et al., 1992; Rice et al., 1986), thereby demonstrating that they cannot be regarded as rudimentary structures. Accordingly, Hüttner et al. (2022) conducted behavioral experiments in which four bottlenose dolphins were tested for electroreception using a stimulus generalization paradigm. All four dolphins responded spontaneously to the first presentation of a weak electric field of 1500 µV cm^−1^ and three of them reliably detected electric field strengths reduced to 500 µV cm^−1^. After these prior data clarified that the bottlenose dolphin must also be considered an electroreceptive species, we examined in this follow-up study the sensitivity of the system by determining detection thresholds for weak electrical DC and AC fields for two bottlenose dolphins. Because any electrosensitive organism can potentially sense a magnetic field (Courtney et al., 2015), we discuss the potential importance – beyond close-range function – of passive electroreception in dolphins for large-scale orientation.

## MATERIALS AND METHODS

### Subjects

The study was conducted with two female bottlenose dolphins [*Tursiops truncatus* (Montagu 1821)] named Dolly and Donna. The dolphins were kept at Nuremberg Zoo, Germany, together with five other dolphins and a group of California sea lions (*Zalophus californianus*). The dolphin enclosure consists of six outdoor pools (dolphin lagoon) connected with an indoor area with three more pools. Experiments were carried out with one animal at a time once a day, generally 5 days per week. During an experimental session, the animals received approximately 20% of their daily diet (1.0–2.0 kg of capelin, herring, sprat and squid). Dolly and Donna had previously participated in a study that demonstrated their ability to detect DC electric stimuli as low as 500 µV cm^−1^ (Hüttner et al., 2022).

All experiments were conducted in accordance with the European Communities Council Directive of 22 September 2010 (2010/63/EU) and the German Animal Welfare Act of 2006. The individuals involved in the study were not subject to pain, suffering or injury; therefore, no approval or notification was required.

### Experimental setup and stimulus generation

**Fig. 1.**
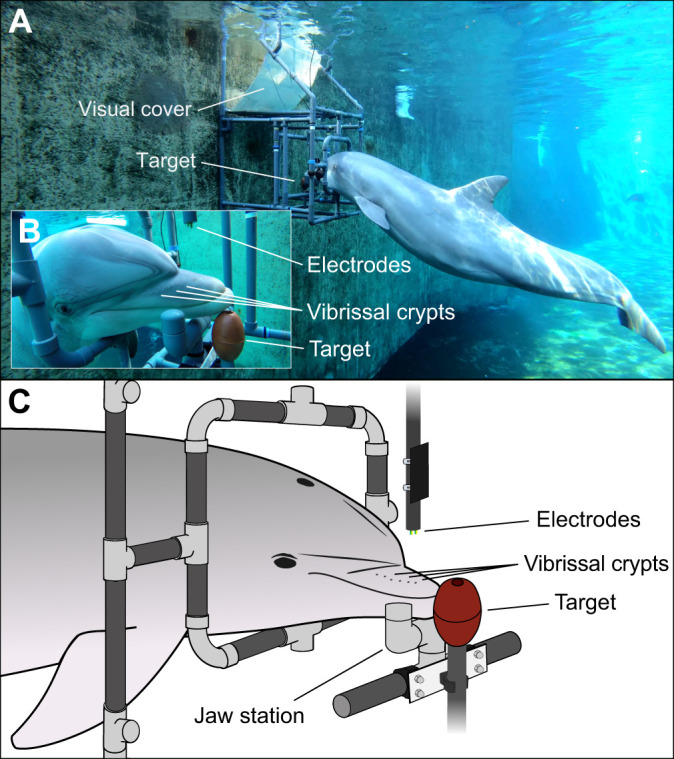
**Overview of the experimental setup during a trial.** (A) Underwater view of a dolphin stationing inside the apparatus during a trial. The dolphin swims head-first into the submerged apparatus and places its rostrum on the jaw station while touching the target with the tip of its rostrum. The visual cover prevents any unintentional cueing by the experimenter as the dolphin cannot see the experimenter as soon as they enter the apparatus. (B) Close-up view of the dolphin stationing inside the apparatus. (C) Close-up schematic view of the experimental setup during a trial. A dolphin stations on the target in the apparatus during a trial. The target and jaw station ensured a constant position of the dolphin's rostrum during all trials. The electrodes are located approximately 10 cm above the hairless vibrissal crypts on the upper rostrum. If an electric stimulus was presented, the dolphin was trained to leave the apparatus (‘hit’) within 3 s after stimulus onset. During stimulus-absent trials, the dolphin remained in station for at least 12 s (‘correct rejection’). Correct responses were secondarily reinforced by the experimenter followed by a fish reward from the trainer. Incorrect responses were not reinforced.

Experiments were conducted in a round indoor pool (diameter 12 m). Sessions were carried out by the experimenter and a trainer who was handling the dolphin between trials. A cubic-formed apparatus was built from PVC tubes and was placed in the pool prior to the start of each session (Fig. 1A). For each trial, a dolphin entered the submerged apparatus through a square opening and touched a target (plastic ball) in the center with the tip of its rostrum (see Fig. 1B,C). Additionally, the dolphins learned to place their lower jaw on a U-shaped jaw station in front of the target to ensure a consistent head position during each trial (see Fig. 1B,C). An underwater camera (WoSports^®^ Fish Finder) attached to the apparatus on the right-hand side of the stationing dolphin was connected to a small monitor screen on land so that the experimenter could observe the dolphin's behavior during each trial. Presentation of the stimulus began when the tested dolphin was resting its rostrum on the jaw station while remaining calmly in the apparatus. A second underwater camera (GoPro Hero 4 Black, GoPro, USA) was used to record all sessions.

To exclude any unintentional cueing by the experimenter, a white visual cover was attached to the apparatus above the dolphin's station (Fig. 1A). The experimenter was sitting on land behind the apparatus, and out of sight of the dolphins as soon as they had entered the apparatus. To eliminate secondary acoustic cues potentially associated with the generation of an electric field, a powerful water jet was directed onto the water surface at a distance of 2 m from the experimental apparatus to create an acoustic masking similar to white noise.

For the generation of weak electric dipole fields, two mechanically stable copper wire electrodes (1 cm long, 2 mm in diameter and 1 cm apart) were placed approximately 10 cm above the dolphins’ rostrum (see Fig. 1C). The location of the electrodes directly in front of the dolphins’ melon prevented the dolphins from seeing them while stationing owing to a blind area above the rostrum (Cozzi et al., 2017; Dral, 1975; Xitco et al., 2004). Furthermore, any secondary cues perceived via echolocation and associated with the onset of an electric field due to electrochemical reactions and subsequent micro-bubbles on one of the electrodes were also considered to be non-detectable. The electrode itself would create a much stronger echo that would overshadow any possible cues caused by the electric stimulus (M. Amundin, personal communication).

The electrodes were connected to a custom-built electric field generator (EFG, version 2.0, 2014, University of Rostock), powered by three 12 V batteries, that acted as a constant current source and created a floating electric circuit. Electric field strength was adjusted using a multi-turn wirewound potentiometer (VISHAY SPECTROL, Model 534, 10 turns, 10 kΩ, VISHAY Intertechnology, Malvern, PA, USA). The stimulus generator was connected to a digital current meter (Voltcraft VC870, Conrad Electronics SE, Germany) to monitor applied current. Either direct current (DC) or alternating current (AC) electric field stimuli were applied by switching the voltage on and off, thus driving the electrodes directly. To exclude any possible polarization effects owing to the usage of copper electrodes as well as impedance differences between on- and off-state, control measurements of the presented electric fields were conducted before and after every session. Despite their lower electrochemical stability, this allowed the use of solid copper electrodes, which show a better resistance against potential contact with the animals and other mechanical impacts. DC stimuli were presented by generating a square wave pulse of adjustable length. Stimulus duration was defined as 3 s based on a timer chip. AC stimuli were presented by generating a square wave periodic signal symmetric to 0 V, using three different frequencies: 1, 5 and 25 Hz. Instead of the more frequently used sinusoidal signals (e.g. Eeuwes et al., 2008; Peters and Evers, 1985), we decided to use square-wave signals, as, for example, in the studies by Dijkgraaf and Kalmijn (1963), Fields et al. (1993) and Kalmijn (1966), because of the better reproducibility using standard timing circuits. The root mean square (RMS) value of square-wave signals is approximately 30% higher compared with sinusoidal signals with the same frequency and amplitude, which could influence the determination of the sensory detection threshold. However, the threshold values determined so far for electric direct and alternating fields in sharks and dolphins are subject to large fluctuations in the absolute values, so no direct influence of signal shape can be derived from this. Moreover, it is unclear whether the RMS value is the critical parameter or, for example, the amplitude, as long as the sensory transduction process in dolphins has not been described. Also, the upper cut-off frequency of the sensory system could be influenced by the signal shape depending on stimulus transduction.

The electric field at the location of the upper jaw of a stationing dolphin was measured before and after each session using two non-polarizable Ag/AgCl electrodes (1 cm long, 0.1 mm thick, 1 cm apart), connected to a custom-built high impedance electric field detector (EFD, version 2.0, University of Rostock). The measurement electrodes were placed in alignment with the stimulus electrodes for maximal voltage drop. To monitor the DC electric field stimulus, the amplifier was connected to a digital voltmeter (Voltcraft VC870, Conrad Electronics SE, Germany) connected to a battery-powered laptop running a recording software via USB (VC 870 Interface Program, Version 4.2.6, Voltcraft, Conrad Electronics SE, Germany). The default recording interval was approximately 2 Hz. AC electric field strength and frequency were measured via a digital oscilloscope (RIGOL DS1052E, Dual Channel, 50 MHz, RIGOL Technologies, Beijing, China) that was connected to the EFD.

We measured the rostrums of both dolphins to the nearest millimeter and determined the position of the vibrissal crypts on the upper rostrum individually for the two test animals. The measurement electrodes were then placed below the stimulus electrodes at the location of the nearest vibrissal crypt to allow for the most precise measurement of the electric field strength reaching the rostrum. Because the dolphins stationed both at the target and at the resting platform, a reproducible exposure of the dolphins’ rostrum in the respective electric field was achieved. Although the construction of the apparatus and the animal training were optimized for threshold determination in stationary dolphins, it must be taken into account that, ultimately, freely behaving animals were the test subjects. Because of attenuation of the electric dipole field with the third power of the distance and the curved field geometry, small distance variances in the centimeter range between the rostrum and the stimulating electrode or small changes in vibrissal crypt orientation within the electric field have significant effects on the electric field value relevant for the animal. Errors here include individual differences in the position of each animal tested, as well as the stimulus transduction to be identified. In this study, the dorsal rostrum surface was exposed to a lateral directed electric field optimal for the stimulation of opposite vibrissal crypts on both sides of the rostrum. If a dorsoventral (along the ampulla-shaped epidermal invagination) or anteroposterior (along unilateral vibrissae crypts) receptor arrangement or receptor circuitry is of greater importance for stimulus reception, strongly divergent absolute threshold values could result. This requires further investigation with correspondingly variable field geometries.

### Experimental procedure

A go/no-go task was used to determine the sensory threshold for DC and AC electric field signals. The dolphins were already trained on this test paradigm in the study by Hüttner et al. (2022). During ‘go trials’ (stimulus-present trials), a correct response (‘hit’) occurred if the dolphin left the station within 5 s of stimulus onset; otherwise, the response was regarded as incorrect (‘miss’). During ‘no-go trials’ (stimulus-absent trials), the correct response was when the dolphin remained in station for more than 12 s (‘correct rejection’). When the dolphin left the station before 12 s, the response was regarded as incorrect (‘false alarm’). All sessions were conducted by a trainer who handled the dolphin, and the experimenter who controlled the apparatus. As soon as the animal stationed properly, the experimenter did or did not present an electric stimulus. The copper electrodes were present at all times so that the only difference between ‘go trials’ and ‘no-go trials’ was the presence or absence of the electric stimulus. During a session, ‘go trials’ and 'no-go trials’ were conducted following a pseudorandom order (Gellermann, 1933). The trainer had no knowledge of whether a ‘go’ or ‘no-go’ trial was being conducted. Observing the dolphins’ responses via the underwater camera, correct responses of the tested animal were secondarily reinforced by an immediate short whistle sound by the experimenter, which was also the sign for the trainer that the tested dolphin's response was correct. The dolphins were then rewarded with fish by the trainer and sent back to the apparatus for the next trial. False responses were immediately signaled by the experimenter with three short repetitive whistle sounds. In this case, the dolphins did not receive a reward from the trainer but were sent to the apparatus for the next trial.

As in the study by Czech-Damal et al. (2012), detection thresholds of the bottlenose dolphins were determined using a combination of the staircase method and the method of constant stimuli, with the threshold defined as the electric field strength the dolphins could still detect in 50% of the trials (Gescheider, 1976, 1997). A predetermined and measured set of stimuli was used in the tests: 500, 250, 125, 100, 75, 50, 40, 30, 20, 10, 7, 5, 3 and 2 µV cm^−1^. For each electric field strength used to approach the threshold of a subject, a minimum of 30 stimulus-present trials were carried out, which were spread over at least three consecutive sessions. Because the detection performance of both dolphins for stimuli whose intensity was well above threshold was almost faultless, further sessions were carried out if their performance fell below 80% correct responses with a newly introduced stimulus strength. In these sessions, the last better-detected stimulus strength was offered first again with the new stimulus intensity interspersed. If a dolphin's performance improved with the previously poorly detected stimulus intensity, three pure sessions with this electric field strength were conducted again with a total of 30 stimulus-present trials, before the next weaker stimulus intensity was introduced. After the threshold determination for DC fields was completed, the two dolphins were tested for their ability to perceive electric AC fields. Test frequencies chosen were 1, 5 and 25 Hz, each tested separately.

## RESULTS

### Sensitivity for DC electric fields

**Fig. 2.**
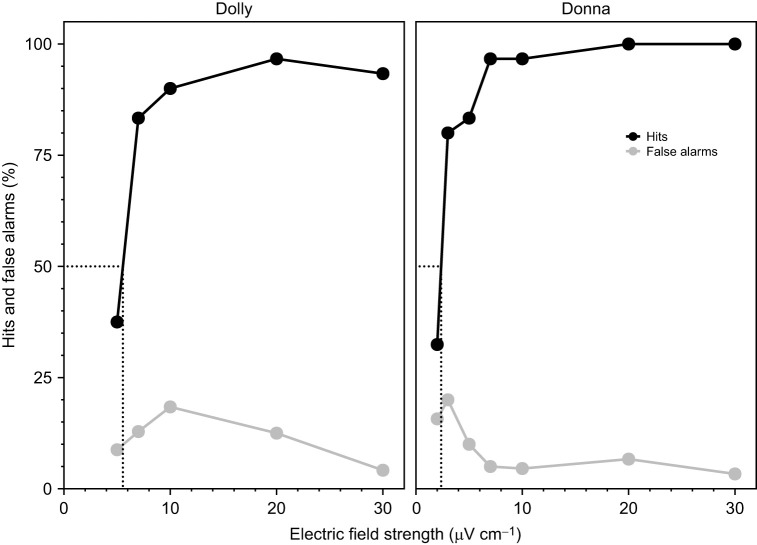
**Results from the psychophysical study with dolphins Dolly and Donna investigating their abilities to detect weak DC electric fields.** Hit rate (%) in relation to stimulus intensity (black) and false alarm rate (%) over all sessions of each stimulus intensity (grey) are shown. Absolute detection thresholds were determined as the signal intensity with a theoretical hit rate of exactly 50.0%: Dolly: 5.5 µV cm^−1^; Donna: 2.4 µV cm^−1^.

Threshold determination for DC electric fields started with an electric field strength of 0.5 mV cm^−1^, the stimulus intensity that both animals formerly detected in >96% of the stimulus-present trials (Hüttner et al., 2022). Dolly achieved 100% correct decisions in 30 stimulus-present trials, while Donna failed to respond only once in 30 trials. Stimulus intensity was then gradually reduced following the predetermined stimulus set. Interestingly, with the first reduction in stimulus intensity, Dolly often started a trial by repeatedly moving her rostrum horizontally from side to side right below the stimulus electrodes before she stationed herself with the tip of her rostrum at the target. Up to a field strength of 125 µV cm^−1^, the hit rate of Dolly was >93%. Here, her performance dropped to 77.7% for the first time, but after a total of seven training sessions she again reached 93.3% hits in the last 30 stimulus-present trials. This improvement in performance during training sessions was almost the same with a field strength of 100 µV cm^−1^ (90% hits in the last 30 stimulus-present trials after eight sessions). With electrical field strengths of 75, 50, 40 and 30 µV cm^−1^, Dolly showed hit rates >83% (93.3%, 86.6%, 83.3% and 93.3%) already in the first 30 stimulus-present trials. Also, with field strengths of 20, 10 and 7 µV cm^−1^, Dolly ultimately achieved high hit rates (96.6%, 90.0% and 83.3%, see Fig. 2), but with these stimulus intensities she required significantly more training sessions (16, 20 and 13, respectively) than before. Correspondingly, with the next weaker stimulus intensity of 5 µV cm^−1^, her hit rate dropped to 37.5% and remained at chance level thereafter. Because Dolly was very reluctant to cooperate at this weak field strength, an intensive training phase could no longer be carried out. Interpolated from her performance at the last electric field strength above threshold (7 µV cm^−1^) and that at the field strength below threshold (5 µV cm^−1^), Dolly's detection threshold at a hit rate of 50% was thus 5.5 µV cm^−1^ (Fig. 2).

With a hit rate between 83.3% and 100%, Donna's performance was very stable over the various stimulus attenuations up to a stimulus strength of 20 µV cm^−1^, which she also detected in 100% of the stimulus-present trials. Up to this electric field strength, her hit rate only fell briefly to chance level (55% hit rate) at 100 µV cm^−1^ but was again >83% after a total of three additional sessions. With a field strength of 10 µV cm^−1^, Donna needed a total of 15 training sessions to finally achieve a hit rate of 96.6% again. At a field strength of 7 µV cm^−1^, her performance dropped only briefly to 66.6%, but was again at 96.6% after only seven training sessions. Donna still detected field strengths of 5 and 3 µV cm^−1^ in 83.3% and 80.0% of the respective last 30 stimulus-present trials conducted, before her performance at 2 µV cm^−1^ dropped to 33.3% and, like Dolly, it became increasingly difficult to motivate her to cooperate. Interpolated from these data, her detection threshold was 2.4 µV cm^−1^ (Fig. 2).

### Sensitivity for AC electric fields

**Fig. 3.**
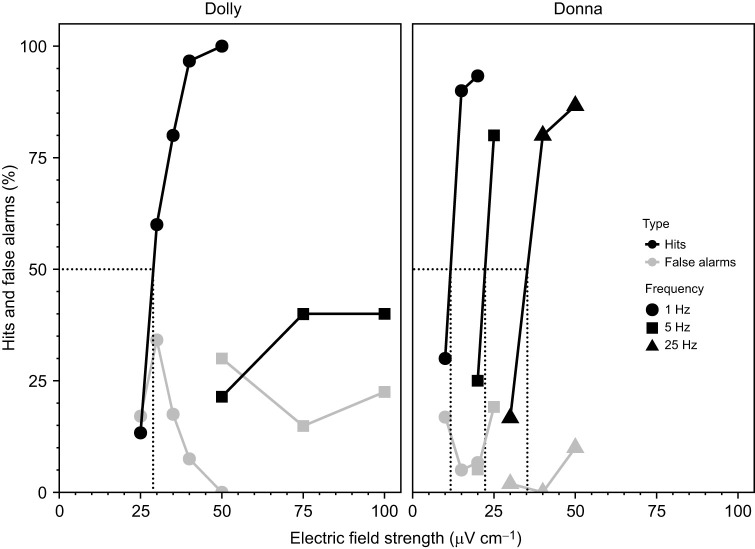
**Results from the psychophysical study with dolphins Dolly and Donna investigating their abilities to detect low frequency AC electric field stimuli of 1 Hz (circles), 5 Hz (squares) and 25 Hz (triangles).** For both dolphins the hit rate (%) in relation to electric field intensity (black) and false alarm rate (grey) over all sessions of each tested electric field intensity for each frequency are shown. The absolute detection threshold for 1 Hz stimuli of Dolly was determined at 28.9 µV cm^−1^ by interpolating the theoretical electric field strength at 50.0% hit rate. Dolly did not respond above chance level after the frequency was set to 5 Hz. Donna still responded well to AC fields of 5 and 25 Hz. Accordingly, the absolute detection thresholds for the three AC frequencies were determined at: 11.7 µV cm^−1^ for 1 Hz stimuli, 22.3 µV cm^−1^ for 5 Hz stimuli, and 35.3 µV cm^−1^ for AC fields of 25 Hz.

At first, 1 Hz AC signals with a stimulus intensity clearly above the previously determined threshold for DC fields were tested. Dolly reached a hit rate of 80% at an AC field strength of 35 µV cm^−1^ before her detection performance fell to 60% correct at 30 µV cm^−1^, and 20 µV cm^−1^ no longer was detected (Fig. 3). Donna still reliably detected 1 Hz electric fields as low as 15 µV cm^−1^ with a hit rate of 90%. However, she could no longer detect the next stimulus attenuation to 10 µV cm^−1^ (Fig. 3). Accordingly, sensory thresholds for 1 Hz AC fields interpolated from these data were 28.9 µV cm^−1^ for Dolly and 11.7 µV cm^−1^ for Donna (see Fig. 3).

After the frequency was set to 5 Hz, Dolly did not respond above chance level anymore, even when the field strength was increased to 100 µV. In contrast, Donna responded well to AC fields of 5 Hz as well as 25 Hz. With a 5 Hz AC field of 25 µV cm^−1^, she achieved a hit rate of 80%. However, after a slight attenuation of the stimulus intensity to 20 µV cm^−1^, her performance dropped to a hit rate of 30%, so that a detection threshold of 22.3 µV cm^−1^ resulted for this stimulus quality (Fig. 3). As Dolly already failed to detect 5 Hz AC fields, stimuli with a frequency of 25 Hz were only tested with Donna. At this frequency, a detection threshold of 35.3 µV cm^−1^ was determined for Donna (Fig. 3).

## DISCUSSION

In the study by Hüttner et al. (2022), it was shown that the vibrissal crypts of bottlenose dolphins are very similar in structure and innervation to those of the electroreceptive Guiana dolphin (*Sotalia guianensis*, Czech-Damal et al., 2012). Following a cognitive approach, the study also demonstrated that four bottlenose dolphins responded to weak electric DC fields. The detection thresholds determined in the present study confirm this result by showing that the absolute sensitivity of bottlenose dolphins to DC electric fields also compares well with that of the Guiana dolphin. While the threshold value determined by Czech-Damal et al. (2012) for the Guiana dolphin was 4.6 µV cm^−1^, the two test subjects in the present study reached thresholds of 5.5 and 2.4 µV cm^−1^, respectively (Fig. 2). Thus, behavioral detection thresholds of the two odontocete species tested so far for electroreception are in the same order of magnitude as those determined in the monotreme platypus (Czech-Damal et al., 2012; Scheich et al., 1986). Starting with the first attenuations of the electric field strength, Dolly repeatedly moved her rostrum horizontally from side to side beneath the stimulus electrodes before stationing on the target, as if searching for an electric stimulus. While foraging, both the platypus and the paddlefish (*Polyodon spathula*) also show movements of their electroreceptive organs in the horizontal plane, potentially to enhance prey detection (Gregory et al., 1989a; Pettigrew and Wilkens, 2003). The fact that in our psychophysical experiments such movements of the rostrum of the dolphins were suppressed by the type of stationing may well have had an impact on the threshold values.

Dolly and Donna also responded well to low-frequency AC electric fields (Dolly 1 Hz only, Donna 1, 5 and 25 Hz), but thresholds for AC fields were generally higher than those determined for DC fields. Although Dolly's detection threshold for a 1 Hz AC signal was 28.9 μV cm^−1^ and thus more than twice Donna's threshold (11.7 μV cm^−1^) for the same frequency, in both animals, the AC threshold for a 1 Hz AC field was higher by a factor of 5 compared with each animal's respective DC threshold. Moreover, the capability of Donna to detect AC electric fields higher than 1 Hz decreased with increasing stimulus frequency. Although Donna was able to detect 5 and 25 Hz signals, she could only do so after the electric field strength had been increased by at least a factor of 2 (see Fig. 3). We have little insight into the physiology of electroreception in toothed whales, but it is interesting that the ratio of detectability of DC and AC fields in dolphins is similar to that in elasmobranchs. Naturally occurring bioelectric fields can be described as dynamic electric fields consisting of a standing DC-dipole electric field modulated by low-frequency AC components arising, for example, from ion exchange processes and gill respiratory movements (Bedore and Kajiura, 2013; Bodznick et al., 1992; Eeuwes et al., 2008; Haine et al., 2001; Kalmijn, 1972, 1974; Wilkens and Hofmann, 2005, 2008). Several studies have shown that sharks and rays respond best to DC electric field signals, but also to low-frequency AC potentials <20 Hz (Eeuwes et al., 2008; Kalmijn, 1971, 1974; Kimber et al., 2011). Rays (*Raja clavata*) only showed good detection capabilities for AC fields of 16 and 32 Hz after increasing the electric field strength by factors of 8 and 32, respectively (Kalmijn, 1974), whereas two shark species (*Scyliorhinus canicula* and *Triakis semifasciata*) no longer responded to AC stimuli with frequencies >16 Hz (Kalmijn, 1973, 1974). Although the ampullary electroreceptors of the elasmobranchs are not DC sensitive, the movement of the animal relative to the stimulus source causes the standing DC electric field signals to be automatically converted into low-frequency AC signals, which represent an adequate stimulus (Bodznick and Montgomery, 2005; Kalmijn, 1974). Taking these relationships into account for the electroreception of toothed whales, tests in which the animals can perceive weak electric fields while moving would be an interesting extension of the present experimental approach.

It has been discussed that passive electroreception in elasmobranchs (Bedore and Kajiura, 2013), the monotreme platypus (Manger and Pettigrew, 1995) and odontocetes (Dehnhardt et al., 2020) may serve the functional role of prey detection during benthic foraging strategies. Bioelectric fields generated by typical prey are in the range of 50­–500 µV cm^−1^ in teleost fish, whereas wounded crustaceans generate even stronger electric fields of more than 1.0 mV cm^−1^ (Kalmijn, 1974). Based on an average detection threshold of 35 nV cm^−1^ obtained from literature data across various elasmobranch species, Bedore and Kajiura (2013) calculated the electric field strength as a function of distance for the electric fields emanating from different prey fish and interpolated from these data a detection range of 0.3–0.7 m for sharks. This underlines empirical data showing that the steep decay of electric potentials with distance from the source even in the highly sensitive elasmobranchs only allow for short-range prey detection (Haine et al., 2001). Taking these calculations into account, thresholds determined for bottlenose dolphins in the present study would indicate that they can detect the same fish species considered for sharks at a distance of 3–7 cm. Although the detection range for weak electric fields of the bottlenose dolphin is thus significantly lower than in elasmobranchs, electroreception could still facilitate benthic prey detection at short distances. In general, toothed whales are able to detect non-visible prey through echolocation. Echolocating dolphins can detect solid objects as small as 8 cm at a distance of more than 110 m (Au and Snyder, 1980) and live fish in their natural habitat at a distance of 93 m (Au et al., 2007). In a benthic feeding strategy, an echolocating dolphin may detect a fish buried 30 cm in the sediment from some distance (Madsen and Surlykke, 2013). Crater-feeding or bottom-grubbing bottlenose dolphins bury themselves deeply into the sea floor to catch fish hidden in the sediment (Mann and Sargeant, 2003; Rossbach and Herzing, 1997; Sargeant and Mann, 2009), where object detection by echolocation is possibly limited owing to reverberation and scattering effects at the sea floor (Au, 1992). As a high degree of water turbidity inevitably results from these benthic foraging strategies, vision toward potential prey should also be maximally occluded (Weiffen et al., 2006). Relying on other modalities, such as electroreception, tactile or hydrodynamic perception, and integrating all sensory information is then important to ensure prey capture (Gardiner et al., 2014; Kelkar et al., 2018; Torres, 2017). Unlike dolphins, sharks initially use their sense of smell instead of sight or hearing to detect and track prey (Hobson, 1963; Hodgson and Mathewson, 1971; Hueter et al., 2004). In attacking their prey, however, electroreception is not only essential for benthic feeding, but also required for successful capture of pelagic prey. In a sensory deprivation experiment with three shark species, Gardiner et al. (2014) found that without the presence of electric cues, the sharks were unable to grab prey even when located directly in front of their mouths. As previously suggested for the Guiana dolphin (Czech-Damal et al., 2012), bottlenose dolphins could also benefit from their electroreceptive capability just prior to prey capture. Although their acoustic senses, including echolocation and passive hearing, as well as their good visual abilities (Herman et al., 1975) are most likely used for initial prey localization, their passive electroreception allows short-range prey detection and the goal-oriented snapping of prey fish in a benthic foraging strategy.

Beyond the role of electroreception in foraging odontocetes, there is good reason to hypothesize that this sensory ability may also support large-scale orientation through use of the Earth's magnetic field. Evidence for magnetoreception in cetaceans has already been drawn from the observation that the location and timing of cetacean live strandings are often associated with geomagnetic anomalies (Kirschvink et al., 1986; Klinowska, 1985, 1986a, 1986b, 1988, 1990) or with perturbations of geomagnetic fields caused by solar storms (Vanselow et al., 2018). The migration routes of fin whales are also suggested to be explainable by a map-like use of the Earth's magnetic field (Walker et al., 1992). Based on the detection of magnetic material, including magnetite in the dura mater of *Delphinus delphis*, it has been suggested that orientation to the Earth's magnetic field in cetaceans may be derived from a magnetite-based system (Zoeger et al., 1981), a mechanism, which, among others (Mouritsen, 2018), is also assumed for other vertebrates (Walker et al., 1997). In behavioral tests, however, bottlenose dolphins showed no (Bauer et al., 1985) or only weak reactions (Kremers et al., 2014) to magnetic stimuli.

Because electric and magnetic fields are closely linked in the marine environment (Newton et al., 2019) and given that any electrosensitive organism can potentially sense a magnetic field (Courtney et al., 2015), it is possible that the ability of dolphins to detect weak electric fields bears the potential for orientation to the Earth's magnetic field through induction-based magnetoreception (Formicki et al., 2019; Johnsen and Lohmann, 2005; Jungerman and Rosenblum, 1980; Lohmann and Johnsen, 2000; Molteno and Kennedy, 2009; Newton et al., 2019). As described for sharks, induction-based magnetoreception means that an animal experiences potential discernible differences induced in its body as it swims through the Earth's magnetic field (Kalmijn, 1974, 1977, 1978, 1981, 1982; Paulin, 1995). For a horizontal component of the Earth's magnetic field of 25 µT, the induced electric field strength at a swimming speed of 1 m s^−1^ is a maximum of 0.25 µV cm^−1^ (Johnsen and Lohmann, 2008). However, owing to linear dependence, a swimming speed of 10 m s^−1^, which has been measured as well within the swim speed range for dolphins (Au and Weihs, 1980; Lockyer and Morris, 1987), would induce an electric field of 2.5 µV cm^−1^. This means that a dolphin might have some control over the perceptibility of the Earth's magnetic field via its swimming speed, e.g. by following a strategy ‘swim faster when higher sensitivity to geomagnetic field lines is required’. Another source of electromagnetic information potentially important for whale orientation may include electric fields induced by salty water masses such as ocean currents or tidal currents moving across the Earth's magnetic field. Such so-called motional electric fields lead to compensating currents with electric field strengths of 0.08 to 8.0 µV cm^−1^ (Newton et al., 2019; Pals, 1982).

Hypotheses regarding a magnetite- or induction-based mechanism for the potential orientation of cetaceans in the geomagnetic field need not be mutually exclusive (Mouritsen, 2018). In sharks, it has also been discussed that both magnetoreceptor systems are involved in orientation to the Earth's magnetic field, using the induction-based system to obtain the compass heading relative to the direction of travel, while a magnetite-based system is useful for detecting anomalies or changes in magnetic field strength (Anderson et al., 2017). The detection thresholds for weak electric fields of the Guiana dolphin (Czech-Damal et al., 2012) and those of the bottlenose dolphins tested in the present study are of a magnitude that seems to indicate the possibility of induction-based magnetoreception. However, it is important to consider open questions such as the effects of the method in which animals are exposed to an electric field, the unknown pathway of stimulus transmission (see Materials and methods), or animal movement in relation to the electric field. Because these effects may impact sensitivity, an expanded approach to understanding electroreception in odontocetes is required. This opens up a new field of research with the potential to find answers to unexplained phenomena, such as the correlation between live whale strandings and geomagnetic anomalies.
